# Synchrotron X-ray imaging of the onset of ultrasonic horn cavitation

**DOI:** 10.1016/j.ultsonch.2022.106286

**Published:** 2022-12-30

**Authors:** Luc Biasiori-Poulanges, Claire Bourquard, Bratislav Lukić, Ludovic Broche, Outi Supponen

**Affiliations:** a Institute of Fluid Dynamics, Department of Mechanical and Process Engineering, ETH Zurich, Sonneggstrasse 3, Zurich 8092, Switzerland; b Silicon Austria Labs GmbH, Villach A-9524, Austria; c European Synchrotron Radiation Facility, CS 40220, Grenoble F-38043, France

**Keywords:** Ultrasonic, Sonication, Cavitation, X-ray imaging

## Abstract

High-power ultrasonic horns operating at low frequency are known to generate a cone-shaped cavitation bubble cloud beneath them. The exact physical processes resulting in the conical structure are still unclear mainly due to challenges associated with their visualization. Herein, we address the onset of the cavitation cloud by exploiting high-speed X-ray phase contrast imaging. It reveals that the cone formation is not immediate but results from a three-step phenomenology: (i) inception and oscillation of single bubbles, (ii) individual cloud formation under splitting or lens effects, and (iii) cloud merging leading to the formation of a bubble layer and, eventually, to the cone structure due to the radial pressure gradient on the horn tip.

## Highlights

• Synchrotron fast X-ray imaging resolves, for the first the time, the dense bubbly liquid volume closely below the horn tip.
• Ultrasonic horn cavitation starts with the cavitation of single bubbles attached to the tip.
• Single bubbles generate individual bubble cloud under splitting or lens effects.
• Clouds merge due to the radial acoustic pressure gradient on the horn tip, and form a cone shape.

## 1 Introduction

Ultrasonic sonicator horns are vibrating probes generating high-amplitude ultrasound in the lower range of the ultrasonic frequency spectrum, typically between 15 and 100 kHz. The power of such high-amplitude ultrasound in liquids lies in the generation of cavitation, which is triggered by the low-pressure phase of the ultrasound waves reaching values below the vapor pressure of the medium. The low pressure or even tension is thereby able to locally rupture the liquid, which results in the nucleation of vapor bubbles. Ultrasonic horn cavitation-induced effects are leveraged in a wide range of liquid processing applications including sonochemistry, homogenization, emulsification, and cell disruption [1], as well as in sonophoresis [2].

Acoustic cavitation is known to result in many bubble structures, as reported in [3] which provides a comprehensive collection of the various bubble morphologies shaped by acoustic cavitation. Ultrasonic horns in liquids have been found to generate a thin bubbly layer attached to the horn surface that acts as a nonlinear thickness resonator, which amplifies and distorts the acoustic wave [4]. As a result, the focusing process, which has been demonstrated to be driven by the radial acoustic pressure gradient on the horn surface [5], shapes the bubble cluster into a cone-like structure. This Conical Bubble Structure (CBS) has been extensively studied around the typical frequency of 20 kHz for a variety of horn diameters and acoustic power. Moussatov *et al.* [6] was the first to identify the CBS for horn diameters of 20, 80 and 120 mm, and attribute it to self-focusing of an attached bubbly layer. Some later studies consistently showed the presence of CBS also for smaller and intermediate horn diameters [7], [8], [9], with Ma *et al.* [10] demonstrating a consistent increase in the cone base diameter and decrease of cone length for increasing acoustic power, and its validity for various horn diameters. Indeed, the conical structure diameter can exceed the horn diameter for high acoustic power, and then behaves as a large cavity whose dynamics follow both acoustic and hydrodynamic properties (*acoustic supercavitation*) [11]. However, the onset of the bubbly layer as well as the phenomenology eventually leading to the conical cloud formation remains unclear. Previous attempts to image the transient cavitation regime leading to the cone structure provided valuable preliminary insights of these transient processes [12], but typically failed, however, to finely resolve them due to limitations inherent to the imaging technique used.

Indeed, conventional imaging (e.g., line-path integrated images or direct visualization) of these processes is challenged by (i) their three-dimensional (3-D) nature, (ii) fluid volume overlapping, (iii) the stochastic spatial distribution of bubble nucleation, (iv) their high speeds and (v) their micrometric scales across a wide depth of view and close to the horn surface. The resulting images are thus mainly blurred, or fail to resolve the 3-D processes. High-speed Xray phase contrast imaging allows to overcome some of the above-mentioned challenges, which has been leveraged in a number of studies looking at ultrasonic cavitation behavior in water as well as in opaque liquid metals [13], [14]. However, no past visualizations, be it with conventional optical or X-ray imaging, have simultaneously provided a sufficient field of view and spatiotemporal resolution to characterize the onset of cavitation bubble structures below an ultrasonic horn.

Here, we report on visualizations performed through this unique imaging technique on a single horn geometry immersed in water at room temperature to elucidate the mechanisms for the cavitation bubble cloud formation, from the early bubbly layer onset to the appearance of the conical structure.

## 2 Experimental methods

**Fig. 1:**
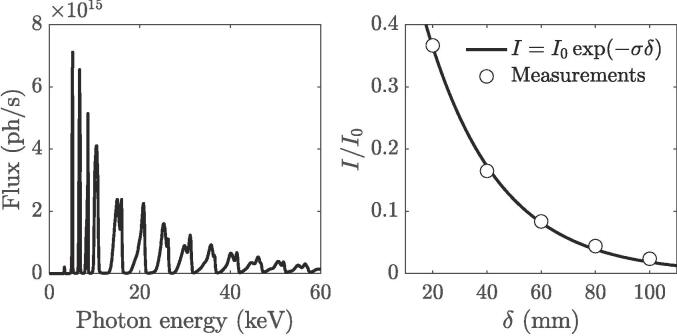
The left graph is a model of the photon flux with respect to photon energy of the ESRF beamline ID19, computed with XOP [16]. The right graph plots the functional dependency of the image intensity over the X-ray path length in water measured from the radiographs (circle markers). The solid line shows the one-term exponential interpolation of the experimental data to determine the absorption coefficient (Beer-Lambert law).

The experiment is carried out at the ID19 beamline of the European Synchrotron Radiation Facility (ESRF) [15], during the 7/8 + 1 filling mode with the integral storage ring current of 200 mA. The hard X-ray pink beam generated by a long-period undulator ($\lambda_{u}$ = 32 mm, total length 1.6 m) set to a 12.5-mm gap is used to probe the fast cavitation dynamics *in-situ*. The generated polychromatic spectrum is only filtered with mandatory optical elements along the 145-m-long vacuum flight tube (2.2-mm-thick diamond window and a series of thin carbon and beryllium windows) to reduce heat load by cutting out the soft part of the X-ray spectrum (<10 keV). The X-ray beam is trimmed upstream of the sample using two sets of in-vacuum slits onto an 8.9 × 7.7-mm^2^ field of view. The characteristic mean energy of the beam is 26 keV with corresponding flux of $1.1\times 10^{12}$ photons/s/0.1 %BW/mm^2^ (see the left graph in Fig. 1). The right graph in Fig. 1 shows the X-ray absorption dependency on the path length in water. A one-term exponential model is fitted to measurements, yielding an absorption coefficient, σ, of −0.037 mm^−1^. The indirect detector assembly, consisting of a 500-μm-thick LuAg:Ce scintillator optically coupled to the Photron SA-Z ultrafast camera with × 2.1 magnification (100:210 Hasselblad tandem optic) with a resulting pixel size of 9.52 μm, is positioned 5 m downstream from the water tank. This ensures that the propagation-based interference between transmitted X-rays results in an increased edge contrast due to (partial) spatial beam coherence properties, while preserving the geometrical representation of imaged phenomena [17]. The experimental arrangement allowed reaching imaging frame rates of 80 kHz with an exposure time of 3.75 μs. Cavitation is generated inside distilled water at the active face of a 1/2″-diameter ultrasonic horn (Branson Ultrasonics Sonifier SFX550, 550 W) operating at 20 kHz. The acoustic radiation direction is from top to bottom. The tip of the horn is immersed 15 mm away from the air–water interface in a 50 × 50 × 50-mm^3^ Plexiglas aquarium. The dimension of the aquarium is selected to reduce the spatial absorption of the X-ray beam by minimizing the path length δ through water. Note that for δ≥ 50 mm, the radiograph signal is no longer exploitable (see Fig. 1, right panel). The input power,$P_{h}$, is selected as percentage values between 20 % and 40 %. The peak-to-peak vertical displacement (µm) of the horn, $d_{n}$, linearly depends on the input power as$d_{n}=1.4P_{h}+7.2$.

## 3 Results and discussion

The radiographs disclose the phenomenology resulting in the formation of a cone-shaped bubble cloud below the ultrasonic horn and indicate a 3-step process: (i) inception of single cavitation bubbles, (ii) formation of individual bubble clouds, and (iii) merging of the clouds to form a bubbly layer and to shape a cone aligned with the horn axis.

**Fig. 2:**
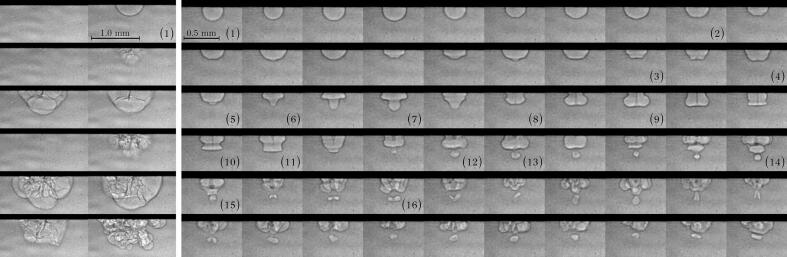
X-ray imaging of the inception and dynamics of single cavitation bubbles at $P_{h}$ = 30 %. The dark region at the top is the tip of the horn, so that acoustic radiation is from top to bottom. The time of frame (1) on the left and right panel is 51.6 ms and 35.5 ms, respectively. The interframe time is 12.5 μs. Note that the transient regime of the ultrasonic horn lasts ≈ 16 ms, after which the full field is established. The Feret diameter of the bubble on frame (1) of left and right panels is 560 μm and 300 μm, respectively. Left and right panels are images from two different experiments.

Upon activation, the ultrasonic horn first results in the inception of single cavitation bubbles attached to the active face of the horn (Fig. 2). The subsequent behavior of the bubble is repeatable and mostly determined by the bubble size. The left panel of Fig. 2 shows the dynamics of a bubble with a diameter, on frame (1), $d_{0}\geq$ 500 μm. The first acoustic cycle from the vapor bubble inception is characterized by a radial oscillation and the complete collapse of the bubble. During the second cycle, the oscillating bubble exhibits pronounced deformation of its interface, which appears as dark discontinuities on the radiographs. A single bubble typically experiences three to five cycles before splitting into numerous daughter bubbles. The dynamics of a bubble with a diameter $d_{0}\leq$ 500 μm is shown on the right panel of Fig. 2, revealing symmetrical intricate bubble shapes. Its motion starts with radial oscillations during a few acoustic cycles, as seen from frame (1), (2), after which it loses its sphericity. The bubble deformation is mostly characterized by successive jet developments and mode shapes. The development of a short jet on the distal wall of the bubble is observed from frame (3), (4a), (4b). The jet changes direction between frame (4) and (5). A larger jet develops during later cycles [frame (8) to (10)] and reaches the horn surface, thus forming a toroidal bubble. The tip of the jet eventually detaches from the distal wall of the bubble but remains attached to the horn surface [frame (10) to (11)]. Simultaneously to the jet retraction, the strong deformations of the bubble surface result in the pinch-off of a daughter bubble [between frame (12) and (13)], which also experiences its own jetting process, as seen from frame (15) to (16). The daughter bubble then experiences successive jetting as seen on the remaining frames. The pronounced deformation of the primary bubble prevents the description of additional jetting processes.

We now discuss the mode shapes disclosed by the radiographs. As time proceeds, the bubble exhibits both zonal and azimuthal modes. In this context, it is instructive to describe the bubble deformation following the mode-shape classification used by [18], which is based on the spherical harmonic terminology and associated (*k*,*l*) modes, where *k* and *l* are the polar and azimuthal mode numbers, respectively.

From frame (5) to (13), the bubble displays strong vertical motion while remaining axisymmetric, which is consistent with the zonal mode shape (*l* = 0). Frames (6) to (7) typically exhibit modes similar to the (6, 0) mode observed by [18]. This mode is accompanied with the development of a jet, as well as the pinch-off of a daughter bubble. Later, the vertical bubble motion and its breakup into two bubbles result in a three-layer structure, clearly visible on frame (14). The layer attached to the horn surface shows horizontal motions resulting in azimuthal perturbations that break the axial symmetry of the bubble, corresponding to the sectoral mode. This layer mode shape is similar to a (4, 4) mode. The identification of the mode shape of the entire bubble volume imaged on frame (14) is, however, challenged by the limited spatial resolution although the images may suggest the presence of a *tesseral* mode (*k*, *l* ≠ *k*). The transition between modes requires additional research efforts, as does the role of the jet trapped inside the bubble in the development of the azimuthal modes.

**Fig. 3:**
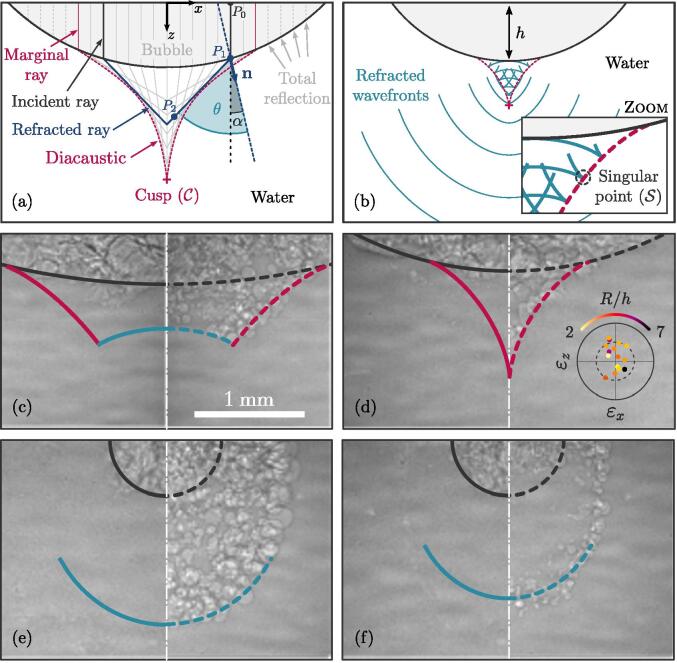
(a) Ray diagram showing the refraction of initially parallel rays incident on and crossing the bubble boundary. (b) Drawing of the refracted wavefronts. Panels (c) to (f) are divided in two parts corresponding to different times. (c) Volcano, (d) cone and (e-f) horseshoe shapes of the observed cavitation bubble clouds ($P_{h}$ = 20 %), overlayed with refracted wavefronts and the diacaustics. The white dashed-dotted line corresponds to the horn axis. The inset in (d) plots the relative error ε on the (*x*,z)-location of the diacaustic cusp between theory and experiments (location of the further-most bubble) where the solid and dashed circles are ε = 10 % and ε = 5 %, respectively. Each circle marker in the inset corresponds to a given experiment, colored with the magnitude of the *R*∕*h* – ratio measured (see arc-shaped colorbar). Note that images on panels (c-d) and (e-f) are extracted from two different test cases, as mentioned in Table 1.

A cloud forming from a single cavitation bubble can originate from two distinct mechanisms: (i) the bubble splits under ultrasound-induced high-amplitude oscillations that overcome the restoring effect of the surface tension and, (ii) the single bubble attached to the active face of the horn acts as an acoustic lens and thus concentrates the acoustic energy to a specific region. For a large impedance ratio resulting in a low energy transfer such as that met in liquid–gas interface problems, past studies have shown that the amplification of the amplitude of the transmitted wavefront can suffice to initiate phase change [19], [20], [21]. The latter mechanism results in characteristic cloud morphologies that agree remarkably well with the time-dependent wave structure shaped by the bubble lens effect. The various resulting shapes for the bubble clouds can be interpreted by applying the classical ray-tracing method to geometrical acoustics. Because the characteristic dimension of the problem is comparable to the acoustic source wavelength, diffraction effects, not considered in this work, may arise. In this approximation, comparison with the experimental observations nonetheless displays reasonable agreement (see the following results and discussion). Within the context of ray theory, the bubble attached to the tip of the horn is modelled as a plano-convex lens of radius *R* and thickness *h*. The characteristic time of the bubble wall deformation is smaller than the time required by the acoustic waves to travel the bubble. The (x − z) plane is defined as the plane of incidence. We let e*_x_* and e_z_ denote the unit vectors in the  *x* and *z* directions, respectively. A rotational symmetry is assumed around the *z*-axis. The inception site of the single bubble giving rise to the cloud is located in (*x*, *z*) = (0, 0). Assuming both vapor and water being homogeneous, rays can be modelled as straight lines along which wavefronts propagate at constant speed. The plane ultrasound wave transmitted from the horn tip to the bubble propagates along a family of parallel rays [see Fig. 3(a)].

**Table 1:** Non-dimensionalized times, with respect to the acoustic period, *T*, of the appearance of the various cloud shapes, as seen in the right-hand sides of Fig. 3(c–f). The time quantity Δ*tf* corresponds to the time interval between the appearance of the cloud structure and the time from which the acoustic field is fully established (determined from horn vertical displacement). Note that the duration of the transient regime of the horn varies from one experiment to another.

Test No.	Shape	*t/T*	Δ*t_f_/T*
1	Volcano	430	116
1	Cone	434	120
2	Horseshoe (filled)	413	125
2	Horseshoe (unfilled)	415	127

We consider the incident ray $P_{0}P_{1}$ as representative of all incident rays. It reaches the spherical wall of the bubble at point $P_{1}$ by making an incidence angle α with the normal **n**.

Trivial geometrical considerations give the coordinates of $P_{1}$ as

(1) $$x_{P_{1}}=Rsin\alpha \;and\;z_{P_{1}}=h-R\left[1-cos\alpha \right]$$

A portion of the incident ray amplitude is reflected at $P_{1}$ into the bubble, while the remaining part of the intensity is transmitted (i.e., refracted) into the bulk water. The refraction occurs for α lower than the critical angle for total reflection, i.e., |α| < α_c_ = arcsin(1/*n*), where *n* is defined as the water–to–air sound speed ratio, *c_w_/c_a_* ≈ 4.4. The refracted ray makes an angle θ with the normal **n** at point $P_{1}$, which is related to α by the fundamental law of refraction θ = arcsin(*n* sin α). To predict the morphology of the cloud from ray theory, we shall derive the parametric equations of the refracted wavefront. We thus consider an arbitrary point $P_{2}$ belonging to the refracted ray resulting from interaction of $P_{0}P_{1}$ with the distal wall of the bubble. The location of $P_{2}$ is given by its position vector **r**_P2_ = **r**_P1_ + **e***_j_*L, where **e***_j_* is the unit vector of $P_{1}P_{2}$, and L is the length of the segment $P_{1}P_{2}$. Letting *t = z_P1_/C_a_* + L /*C_w_* denote the time required to travel from $P_{0}$ to point $P_{2}$ yields L = (*C_a_t-Z_P1_*). Substituting the expression of L into the equation for **r**_P2_, and expressing the unit vector of $P_{1}P_{2}$ as **e**_j_ = **e**_z_ cos(θ − α) − **e***_x_* sin(θ − α), the $P_{2}$-coordinates in Cartesian components read

(2) $$x_{P_{2}}=Rsin\alpha -n\left(c_{a}t-z_{P_{1}}\right)sin\left(\theta -\alpha \right)$$

(3) $$z_{P_{2}}=h-R\left[1-cos\alpha \right]+n\left(c_{a}t-z_{P_{1}}\right)cos\left(\theta -\alpha \right)$$

Eqs. (2), (3) are the parametric equations (parameterized by α) for the location of the refracted wavefront at time *t*. As evidenced in Fig. 3(b), each refracted wavefront (light blue lines) exhibits a singular point S converging towards the *z*-axis as time proceeds. The refracted wavefront therefore consists in two segments, linked at S, where each segment corresponds to a family of refracted rays defined on two distinct α-domains. We refer the reader to Ref. [21] for more details on the occurrence of such a singular point S. Considering the rotational symmetry, S traces out a surface which represents the envelope of the refracted rays. This surface is known as the caustic by refraction, the so-called *diacaustic* [22]. Following previous works [23], [21], the parametric equations for the diacaustic of the bubble, normalized with respect to *R*, can be written as

(4a) $$\tilde{x}\left(\alpha \right)=n^{2}{sin}^{3}\alpha$$

(4b) $$\tilde{z}\left(\alpha \right)=\frac{h}{R}-1+\frac{n^{2}{cos}^{3}\alpha +n{\left(1-n^{2}{sin}^{2}\alpha \right)}^{\frac{3}{2}}}{n^{2}-1}$$

The focal of the bubble $f=\lim_{\alpha \to 0}\tilde{z}\left(\alpha \right)$ corresponds to the cuspidal point C of the diacaustic, where S vanishes. The coordinates of C can be estimated in the limit α → 0 as $x_{C}=0$ and $z_{C}=h+R/\left(n-1\right)$. Plotting the refracted wavefront for different times, along with the diacaustic, reveals three structures: the volcano [Fig. 3(c)], the cone [Fig. 3(d)] and the horseshoe shapes [Fig. 3(e-f)]. The lefthand side of [Fig. 3(c-f)] shows the initial bubble (perturbed but continuous^1^) before the formation of the cloud, which is displayed on the right-hand side. Individual bubbles are distinguished from corrugations by tracking closed contours. In the absence of multiple closed contours, dark discontinuities reveal corrugations at surface of a continuous individual bubble. Note that it is also instructive to examine the location of the gas volume collapse to distinguish individual bubbles from clusters. When a bubble cloud collapses, the individual bubbles constituting the cloud all collapse towards their own center and not toward the center of the cloud. Here, the gas volume we assumed to be continuous collapse towards the center of the apparent volume. The time interval between the left- and right-hand side, divided by the dashed white line, of Fig. 3(c-e) is 25.0 μs and Fig. 3(f) is 37.5 μs. The superposed wavefront and diacaustic on Fig. 3(c-f) are computed from Eqs. (2), (3), and (4), where *R* and *h* are determined by fitting a sphere on the contour of the bubble shown on the left-hand side (dark solid line) extracted using an edge detection algorithm. The time of the computed wavefronts is selected to match the experimental observations of the bubbles, where the time shift is explained by the delay associated with bubble growth. The volcano-like shape corresponds to the early stage of the cloud development as the refracted wavefront still experiences convergence. The bowl-shaped top of the volcano is drawn by the refracted wavefront while its sides are traced out by the diacaustic. The conical shape is the transitional stage where the convergence of the refracted wavefront is completed. The cone is fully delimited by the diacaustics. The horseshoe is a later structure as it appears for a diverging refracted wavefront beyond the focal distance of the bubble. The filled or unfilled nature of the horseshoe shape is assumed to be determined by the bubble oscillation time offset along the direction of the wave propagation. Distal bubbles are the last to collapse [Fig. 3(f)]. With no experimental access to the liquid volume under the horn, the acoustic pressure amplitude cannot be measured. However, estimating the peak pressure to be 1.5 bar and the size of the pre-existing nuclei to be about 5 μm [5], the maximum bubble radius is reached at 24.4 μs and the first collapse at 30.5 μs according to the Rayleigh-Plesset equation, which are consistent with the times corresponding to Fig. 3(c-f).

**Fig. 4:**
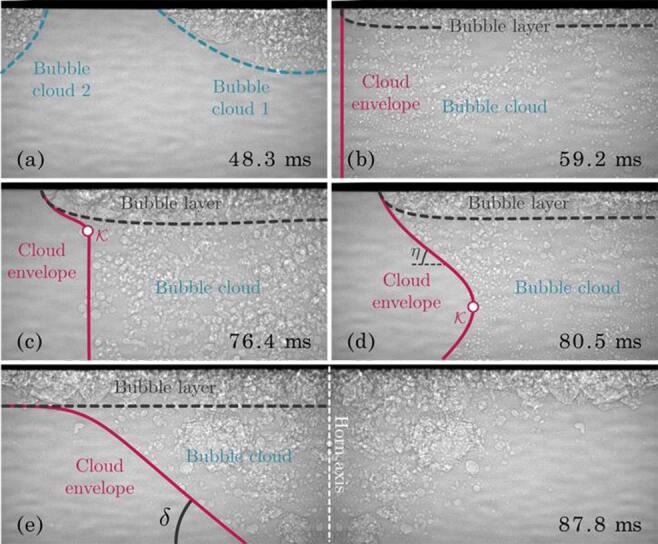
Phenomenology of the conical cloud formation at $P_{h}$ = 40 %. The right-hand side corresponds to the horn axis. (a) Individual clouds. (b) Cylindrical cloud and bubble layer. (c) Appearance of a kink on the cloud envelope. (d) Traveling of the kink and formation of the hourglass shape. (e) Mirrored image of the cone-shaped cloud. The image width is 6.0 mm.

Fig. 4 displays the time history of the conical cloud formation. It unprecedentedly reveals that the conical shape is not instantaneous, but is instead preceded by various morphologies. The individual clouds developed from the single bubbles grow and merge by creating a thin bubbly layer [Fig. 4(a-b)]. Note that the various individual bubble clouds eventually constituting the bubbly layer emerged from different individual cavitation bubbles at different locations. The bubble layer is accompanied with the formation of a bubbly cloud delimited by the edge of the bubble layer [Fig. 4(b)]. Considering the axial symmetry of the system, both the layer and the cloud are a bubble column with diameter and height (*d_l_*, *h _l_*) and (*d_c_*, *h*), respectively. At the early stage, *d_l_ = d_c_* and *h _l_* ≪ *h*_c_. As time proceeds, the cloud envelope is characterized by a kink κ with coordinates ($x_{k}$, $z_{k}$) [Fig. 4(c)], which is initially attached to the bubble layer distal wall and reduces the diameter of the bubble cloud so that *d_l_* > *d_c_*. The kink moves away from the bubble layer by converging towards the horn axis. This κ-motion deforms the cylindrical cloud envelope into an hourglass shape, as seen in Fig. 4(d) (red line), so that *d_c_* = *d*(t, *z*) depends on time and *z*. The bubble cloud eventually shapes a cone when $x_{k}\to 0$, observable in Fig. 4(e) despite the limited field of view. Note that the slope η of the upper-half of the hourglass shape remains constant as κ propagates towards the horn axis, which eventually leads to δ = η, where δ is the cone angle [see Fig. 4(e)]. The deformation of the cloud from a cylindrical bubbly column to a cone-like shape is consistent with the previously reported focusing process, which results from the radial acoustic pressure gradient on the horn tip at the origin of a radial sound speed gradient [5].

## 4 Conclusion

We operated high-speed radiography to resolve the onset of horn cavitation occurring close to the horn tip, unreachable with conventional imaging. We demonstrated the ability of radiographs to overcome two limitations inherent to conventional imaging in particular: (i) their limited depth of field which prevents the in-focus image of the bubble activity due to the stochastic nature of the location of their nucleation sites, and (ii) the signal line-path integration preventing the detection of bubble overlapping. Radiographs do not suffer from these limitations by taking advantage of a virtual infinite depth of field and by resolving interfaces between two phases. Radiographs revealed that the cone-shaped bubble cloud results from a 3-step process, starting with the growth of single cavitation bubbles. Complementing X-ray images with ray theory, we showed that bubbles experiencing nearly radial oscillations generate bubble clouds due to the lens effect resulting from the bubble curvature and the water-to-air sound speed mismatch. Deformed bubbles form bubble clouds by splitting when the amplitude of their oscillations overcomes the repelling force of the interface. The clouds eventually merge to form a bubbly layer at the origin of a unique bubble cloud. As time proceeds, the radial pressure gradient on the horn tip successively shapes the cloud into a cylinder, an hourglass, and finally, a cone. Repetitions of the experiments with same initial conditions show the phenomenology reported herein to be repeatable and independent of $P_{h}$. In this work, we addressed the bubble lens effect at the origin of the cavitation bubble cloud by assuming the pressure resulting from the focusing to be sufficient to generate cavitation. However, quantifying the pressure field below the initial bubble attached to the horn tip should be considered in future works to validate the focusing process hypothesized here.

## CRediT authorship contribution statement

**Luc Biasiori-Poulanges:** Conceptualization, Methodology, Formal analysis, Investigation, Data curation, Writing – original draft, Writing – review & editing, Visualization, Funding acquisition. **Claire Bourquard:** Conceptualization, Methodology, Investigation, Data curation, Writing – review & editing, Visualization. **Bratislav Lukić:** Methodology, Investigation, Resources, Writing – original draft, Writing – review & editing, Funding acquisition. **Ludovic Broche:** Methodology, Investigation, Resources, Writing – review & editing, Funding acquisition. **Outi Supponen:** Conceptualization, Investigation, Resources, Data curation, Writing – review & editing, Visualization, Supervision, Project administration, Funding acquisition.

## Declaration of Competing Interest

The authors declare that they have no known competing financial interests or personal relationships that could have appeared to influence the work reported in this paper.

## Figures and Tables

**Fig. 5:**
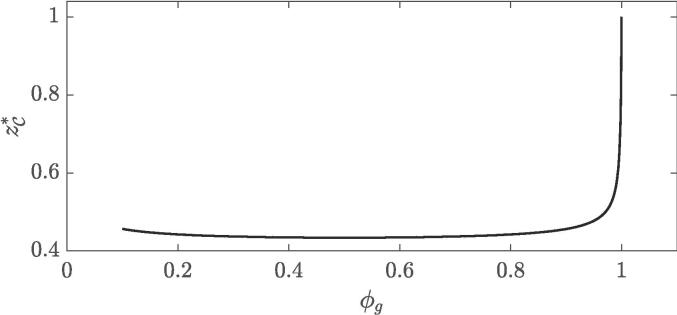
Functional dependency of the cuspidal location ${z_{C}}^{*}$ on the gas volume fraction for R = 1 mm and h = 0.2R.

## Data Availability

Data will be made available on request.

## Appendix A Lens effect of bubbly liquids

As mentioned earlier, we assumed the initial bubbles which eventually result in the clouds observed on Fig. 3 to be perturbed but continuous. It is challenging to precisely determine from the images whether the initial gas volume is corrugated but continuous or consists rather of a bubbly liquid. While our observations seem to indicate a single continuous bubble, where the perturbations appear as blurred lines as opposed to interfaces which appear sharp and this being clearer in the movies compared to static images, we here provide a discussion on the possibility of bubbly liquid, i.e., a liquid–gas mixture, at the origin of the focusing. The sound speed of such a mixture, $c_{m}$, is given by the Wood speed of sound [24], [25], which depends on the density, the sound speed and the volume fraction of each phase involved. Assuming a bubbly water plano-convex lens, n reads $c_{w}/c_{m}$.

Even if we observed a bubbly liquid rather than a perturbed but continuous bubble, radiographs would still not allow for the determination of the gas volume fraction $\phi_{g}$. To evaluate the sensitivity of the focusing on $\phi_{g}$ we nonetheless computed the cuspidal $z_{C}$-location by assuming initial bubbly liquids with $\phi_{g}=\left[0,1\right]$. We denote ${z_{C}}^{*}={z_{C}}^{m}/{z_{C}}^{b}$, where ${z_{C}}^{m}$ and ${z_{C}}^{b}$ are the $z_{C}$-location for a bubbly liquid and a homogenous bubble, respectively. Fig. 5 plots the functional dependency of ${z_{C}}^{*}$ on the gas volume fraction $\phi_{g}$. Not surprisingly, ${z_{C}}^{*}=1$ when the bubbly liquid consists of gas only ($\phi_{g}$ = 1), and then strongly decreases (up to ≈55%) as the volume fraction of water increases. Note that adopting the bubbly liquid assumption requires a very precise determination of $\phi_{g}$ – non achievable based on our images – as ${z_{C}}^{*}$ strongly varies (from 1 to 0.5) for $\phi_{g}$ ranging from 0.97 to 1.0.

## Footnotes

1 See Appendix A for a detailed discussion.
